# Type 2 diabetes reversal with digital twin technology-enabled precision nutrition and staging of reversal: a retrospective cohort study

**DOI:** 10.1186/s40842-021-00134-7

**Published:** 2021-11-15

**Authors:** Paramesh Shamanna, Shashank Joshi, Lisa Shah, Mala Dharmalingam, Banshi Saboo, Jahangir Mohammed, Maluk Mohamed, Terrence Poon, Nathan Kleinman, Mohamed Thajudeen, Ashok Keshavamurthy

**Affiliations:** 1 Twin Health, Bangalore, Karnataka 560043 India; 2 Twin Health, Mountain View, California, 94043 USA; 3 Bangalore Endocrinology & Diabetes Research Centre, Bangalore, Karnataka 560003 India; 4 Diacare – Diabetes Care & Hormone Clinic, Ahmedabad, Gujarat 380005 India; 5 Kleinman Analytic Solutions, LLC, Missouri City, TX 77459 USA

**Keywords:** Type 2 diabetes, Diabetes reversal, Precision nutrition, Artificial intelligence, Diabetes medication elimination, Digital twin technology

## Abstract

**Background:**

Type 2 diabetes reversal has been viewed in the literature primarily as a dichotomous event (reversed or not reversed), even though this viewpoint may not be optimal for clinicians or patients. This cohort study’s objectives were to define stages of type 2 diabetes reversal and measure changes in reversal stages before and after 90 days of digital twin-enabled precision nutrition therapy.

**Methods:**

This study defines seven stages of diabetes reversal. The study is a retrospective pre/post comparison of changes in reversal stage, hemoglobin A1c (HbA1c), weight, body mass index (BMI), and other metrics measured before and after precision nutrition therapy. Reversal stages were defined as Stage 0: HbA1c < 5.7% without medication for > 1 year, Stage 1: HbA1c < 5.7% without medication for < 1 year, Stage 2: HbA1c < 6.5% without medication, Stage 3: estimated HbA1c (eA1c) between 5.7 and 6.4% without medication, Stage 4: estimated HbA1c (eA1c) between 5.7 and 6.4% with metformin monotherapy, Stage 5: dual oral therapy, Stage 6: > = 3 medications.

**Results:**

Reversal stage information was available for 463 patients at baseline and 90 days. At baseline, the proportions of patients in each reversal stage were Stages 1 and 2: 0%, Stage 3: 1%, Stage 4: 8%, Stage 5: 6%, and Stage 6: 85%. After 90 days, the proportions in each reversal stage were Stage 1: 2%, Stage 2: 9%, Stage 3: 32%, Stage 4: 39%, Stage 5: 7%, and Stage 6: 11%, indicating significant progress. Reversal stage progression rates varied by patient subgroup.

**Conclusions:**

Type 2 diabetes patients reached differing reversal stages during 90 days of precision nutrition therapy. Use of reversal stages may benefit patients during therapy.

**Trial registration:**

This was a retrospective study that was approved by the Medisys Clinisearch Ethical Review Board (without registration number) in 2019.

## Background

Until the past decade, type 2 diabetes was seen primarily as a chronic, incurable disease where therapy goals focused on reducing symptoms and slowing disease progression. That viewpoint has begun to change. In 2009 the American Diabetes Association (ADA) discussed diabetes remission in a consensus statement [1]. In its 2016 global report on diabetes, the World Health Organization indicated that diabetes reversal can be achieved [2]. During this time, a number of studies have examined the impacts of very-low-calorie diets, low carbohydrate diets, and bariatric surgery on achieving diabetes reversal [3].

Some variance exists in the definitions of reversal used. The 2009 ADA consensus statement defined complete diabetes remission as a return to normal HbA1c or fasting glucose < 100 mg/dl for at least a year with no pharmacologic therapy or ongoing procedures [1, 4]. The statement also defined partial remission as achieving sub-diabetic hyperglycemia (HbA1c < 6.5%, fasting glucose < 125 mg/dl) for a year without pharmacologic therapy or ongoing procedures [1, 4]. A more recent 2021 ADA consensus statement defined diabetes remission as HbA1c < 6.5% at least 3 months after stopping glucose-lowering pharmacotherapy [5]. Other studies have defined remission using various similar thresholds [3, 6–8]. Consideration has also been given to excluding metformin from the hypoglycemic treatments in the definition of remission or reversal [9].

Despite the existence of definitions for both partial and complete reversal, the primary endpoint in most studies was a dichotomous result such as reversed vs. not reversed (or reached remission vs. did not reach remission). Stages of diabetes reversal have not been thoroughly defined or examined.

Prior research has shown that type 2 diabetes is a heterogeneous disease encompassing four subgroups with distinct pathophysiology and risk of complication [10]. These four subgroups have been described as severe insulin-deficient diabetes (SIDD), severe insulin-resistant diabetes (SIRD), mild obesity-related diabetes (MOD), and mild age-related diabetes (MARD).

The objectives of this study are to define stages of type 2 diabetes reversal and measure changes in reversal stages before and after digital twin-enabled precision nutrition therapy. This is the first such study, and it was hypothesized that reversal stage progression would occur within the first 90 days of therapy and that progression would occur at varying rates across distinct subgroups of type 2 diabetes.

## Methods

### Study design and patient population

This study defines seven stages of diabetes reversal. The study is an observational retrospective pre/post comparison of changes in reversal stage and other metrics within type 2 diabetes patients in India. These patients voluntarily enrolled with consent in the Twin Precision Nutrition (TPN) Program between November 2018 and June 2020 for diabetes therapy. Program inclusion criteria included type 2 diabetes patients with adequate hepatic function (aspartate transaminase or alanine transaminase <= three times the upper limit of normal) and adequate renal function (serum creatinine <= 1.5 mg/dl or estimated glomerular filtration rate > 60 mL/min/1.73m^2^). Exclusion criteria included history of ketoacidosis, diagnosis of major psychiatric disorders, or occurrence of myocardial infarction, stroke, or angina within the last 3 months prior to enrollment. This study was approved by the Medisys Clinisearch Ethical Review Board (without number), and all research was in compliance with the World Medical Association’s Declaration of Helsinki. Data are available from the authors upon reasonable request.

### Twin precision nutrition program

The outpatient TPN Program uses Whole Body Digital Twin technology, powered by artificial intelligence and Internet of Things, to understand the patient’s unique metabolic impairment. The platform collects daily data from continuous glucose monitors (CGM), sensor watches, blood pressure meters, smart scales, detailed patient food intake information, and a mobile app to track and analyze the body’s health signals in order to personalize the patient’s treatment and provide daily precision nutrition guidance to the patient. The TPN platform uses artificial intelligence technologies to build a digital twin model of the patient with biological data from sensors and bloodwork and nutritional data from the TPN mobile app. This digital twin is a dynamic representation of the patient’s specific metabolism, allowing personalized prediction of future health states for different interventions and the selection of the ideal intervention for that patient. This includes nutrition, exercise, and sleep recommendations. In the app, participants input each food item and its quantity from every meal consumed by selecting it from a database of more than 50,000 foods and full nutritional values. Machine learning algorithms analyzed the patient’s macronutrients, micronutrients, and biota nutrients to measure and predict glucose response to specific foods. Participants were provided with specific daily food recommendations to avoid glucose spikes. Calorie consumption was not capped, and patients were allowed to consume food ad-libitum to satiety. Nutritional counselling was provided by trained health coaches through the app and via telephone. Physicians provided daily supervision of patients’ symptoms, CGM values, and diabetes medications. Patient insulin use was adjusted by physicians based on average daily blood glucose levels. Physicians provided and titrated metformin and dipeptidyl peptidase-4 inhibitors over time as blood glucose levels improved. Medication management by physicians was based on customized guidelines. Additional TPN Program detail has been published previously [11].

### Outcome measures

**Fig. 1:**
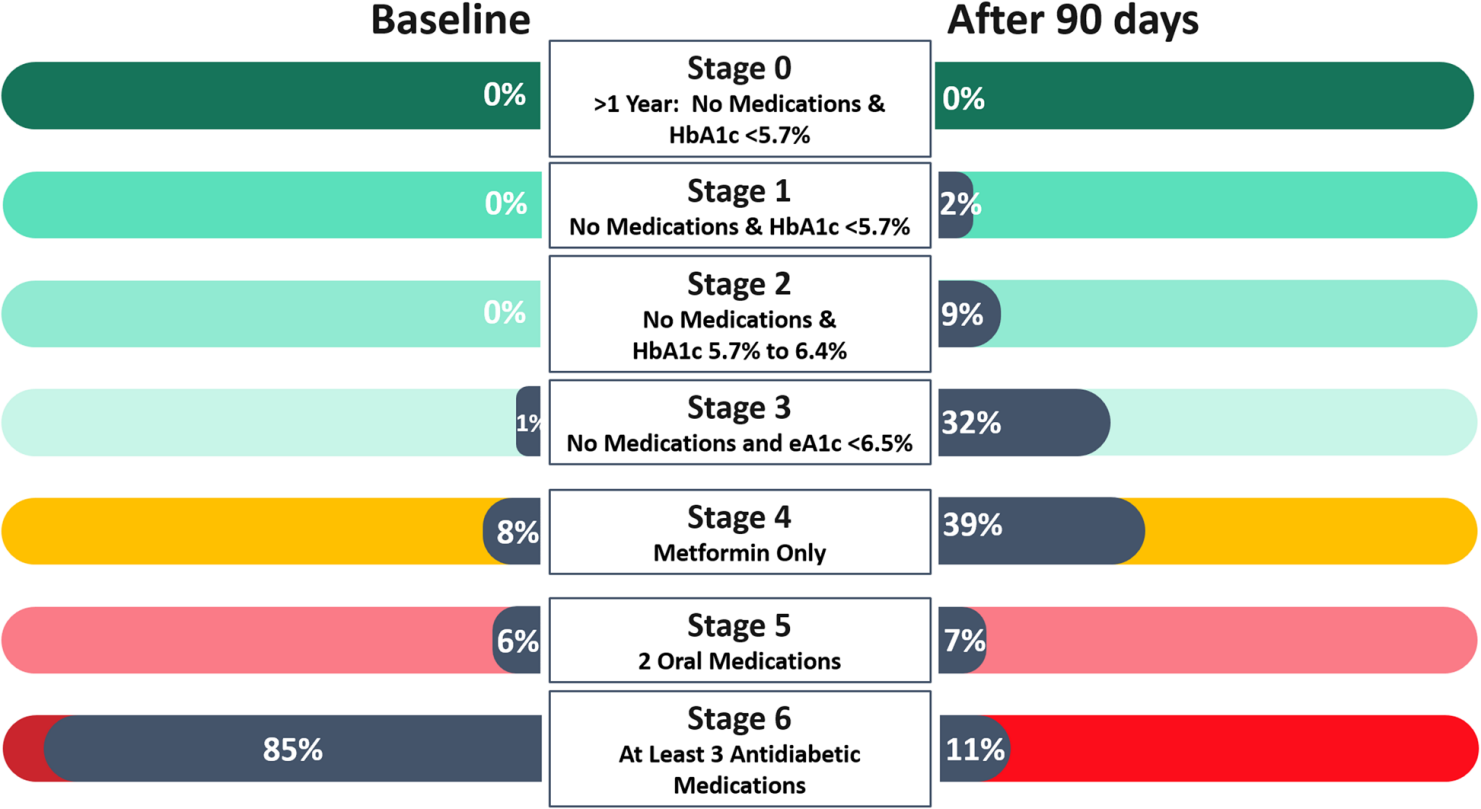
Percent of 463 Patients in Stages of Diabetes Reversal at Baseline and 90 Days. HbA1c: glycated hemoglobin. eA1c: estimated glycated hemoglobin from continuous glucose monitoring

Until now diabetes reversal has most often been considered binary, with a patient having reached reversal or not. Because diabetes reversal involves the correction of complex pathophysiologies, considering reversal in stages would be ideal clinically and motivating for the patient. Hence we propose staging diabetes reversal as follows: at least three antidiabetic medications (Stage 6), dual oral therapy (Stage 5), metformin monotherapy (< 2000 mg) with estimated HbA1c (eA1c, based on CGM measurements) between 5.7 and 6.4% (Stage 4), no antidiabetic medication with eA1c between 5.7 and 6.4% (Stage 3), HbA1c < 6.5% without antidiabetic medication (Stage 2), HbA1c < 5.7% without antidiabetic medication for less than 1 year (Stage 1), and HbA1c < 5.7% without antidiabetic medication for more than 1 year (Stage 0) (Fig. 1).

In order to determine if progression through reversal stages varied within subgroups of patients, the baseline values of six variables were used to classify subgroups of type 2 diabetes patients into four clusters: hemoglobin A1c (HbA1c, %), alanine aminotransferase test values (ALT, u/L), homoeostatic model assessment 2 estimates of β-cell function (HOMA2-B) and insulin resistance (HOMA2-IR), body mass index (BMI, kg/m^2^), and age at onset of diabetes.

The study examined two main outcomes corresponding to two specific hypotheses. The primary outcome was the change in distribution of patients across reversal stages from baseline to 90 days of participation in the TPN Program. The first hypothesis was that the distribution of patients across stages of diabetes reversal would significantly change over the initial 90 days, with progression from higher to lower reversal stages. A chi-square power analysis with power equal to 80%, significance level of 0.05, and medium Cohen’s effect size (0.3) suggested the need for at least 152 patients to detect a change in proportion of patients across the seven reversal stages. A related outcome that was examined was the percent of patients in each type 2 diabetes cluster who reached reversal stage 3 by 90 days. It was hypothesized that the percent of patients reaching stage 3 within 90 days would vary significantly by cluster. A chi-square power analysis with power equal to 80%, significance level of 0.05, and medium Cohen’s effect size (0.3) in this case suggested the need for at least 122 patients to detect a difference across clusters in the proportion reaching stage 3 by 90 days.

Some additional secondary measures were available at baseline and at 90 days for approximately half of the patients. Since these additional measures were not available for all patients, they are included here for descriptive purposes only. They included triglycerides (mg/dL), high density lipoprotein (HDL, mg/dL), very low density lipoprotein (VLDL, mg/dL), apolipoprotein B (ApoB, mg/dL), gamma-glutamyltransferase (GGT, u/L), weight (kg), creatinine (mg/dL), estimated glomerular filtration rate (eGFR, mL/min/1.73m^2^), testosterone (ng/dL), total white blood cell (WBC) count (cells/mm^3^), platelet count (1000/mm^3^), albumin (g/dL), globulin (g/dL), sodium (mEq/L), potassium (mEq/L), magnesium (mEq/L), chloride (mEq/L), and 10-year atherosclerotic cardiovascular disease (ASCVD) risk score [12].

### Statistical analysis

A k-means cluster analysis was used to define four subgroups of characteristically similar patients using HbA1c, HOMA2-B, HOMA2-IR, ALT, BMI, and age at onset of diabetes. Patients were removed from the cluster analysis and subsequent calculations if their values of HbA1c, HOMA2-B, HOMA2-IR, ALT, BMI, and age at onset of diabetes were considered outliers (more than five standard deviations above or below the mean). A Kaplan-Meier survival analysis was performed to compare the number of days from a patient’s enrollment to reaching diabetes reversal Stage 3 (discontinuance of all antidiabetic medications and eA1c < 6.5%) between clusters. A log-rank test was used to determine significant differences between pairs of survival curves. Additionally, chi-square tests were used to assess differences in the percent of patients reaching Stage 3 within 90 days across clusters. A Wilcoxon signed rank test with continuity correction was used to test the significance of the change in reversal stage distribution for all patients from baseline to 90 days. For a subset of patients with additional available data, baseline vs. 90-day comparisons were made for several additional descriptive variables. Continuous variables were described using medians or means and standard deviations. Percentages were used to report means of binary variables. Changes in mean values of continuous variables from baseline to 90 days after enrollment were assessed using paired t-tests. When a particular metric was missing for some patients, the number of patients included in the calculation are noted, and patients with missing values were removed from paired pre-post *p*-value calculations. Analyses were performed using IBM SPSS Statistics for Windows, version 27 (IBM Corp., Armonk, NY), RStudio  version 1.4.1103 RStudio Team 2021 (RStudio: Integrated Development for R. RStudio, PBC, Boston, MA). Microsoft Excel, 2007 (Microsoft Corporation, Redmond, WA), and Google Sheets, 2020 (Google LLC, Mountain View, CA).

## Results

Of 485 patients in the TPN Program, ten were excluded from the analysis due to outlier HOMA2-B values (3 patients), due to outlier HOMA2-IR values (2 patients), due to an outlying BMI value (1 patient), due to outlier ALT values (3 patients), or due to having age at onset of diabetes less than 18 (1 patient). Clusters were defined for the remaining 475 patients. At program enrollment the mean (standard deviation, SD) age for the 475 patients was 48.6 (10.6) years, time since onset of diabetes was 9.2 (7.6) years, HbA1c was 9.0 (1.9), and 130 patients (27.4%) were female. Twelve of the 475 patients did not have reversal stage information available at baseline and 90-days. The remaining 463 patients’ age was 48.5 (10.6) years, time since onset of diabetes was 9.1 (7.5) years, HbA1c was 8.9 (1.9), and 127 (27.4%) were female.

The clusters (Table 1) defined in this study closely resembled the four type 2 diabetes clusters identified by Ahlqvist et al. in a Swedish patient study [10] and the four clusters identified by Anjana et al. in a population of patients in India [13]. Cluster 1 had high HOMA2-B, HOMA2-IR, and BMI and low HbA1c and was designated Mild Obesity-related Diabetes (MOD). Cluster 2 had high age at onset and low HbA1c and was termed Mild Age-Related Diabetes (MARD). Cluster 3 had high ALT and high HOMA2-IR and is classified as Severe Insulin-Resistant Diabetes (SIRD). Cluster 4 had high HbA1c, low HOMA2-B, and low age at onset and was classified as Severe Insulin-Dependent Diabetes (SIDD).

**Table 1:** Type 2 Diabetes Baseline Cluster Characteristics

		1-MOD (*N* = 64)	2-MARD (*N* = 160)	3-SIRD (*N* = 83)	4-SIDD (*N* = 156)	Total (*N* = 463)
Age at Onset (y)	Mean (SD)	41.3 (8.5)	44.6 (7.9)	36.0 (7.0)	35.2 (6.8)	39.4 (8.6)
Duration of Diabetes (y)	Mean (SD)	7.1 (6.5)	9.0 (7.4)	7.2 (6.0)	11.1 (8.3)	9.1 (7.5)
HbA1c (%)	Mean (SD)	8.1 (1.7)	7.8 (1.2)	9.0 (1.5)	10.5 (1.7)	8.9 (1.9)
HOMA2-B	Mean (SD)	110.0 (52.4)	61.3 (29.3)	52.8 (28.9)	27.0 (15.1)	54.9 (39.9)
HOMA2-IR	Mean (SD)	3.9 (1.9)	1.5 (0.7)	2.2 (1.1)	1.7 (1.1)	2.0 (1.4)
BMI (kg/m^2^)	Mean (SD)	33.4 (6.0)	27.4 (3.8)	27.2 (4.3)	26.6 (4.0)	27.9 (4.9)
Weight (kg)	Mean (SD)	88.6 (14.8)	74.8 (12.3)	79.2 (14.4)	74.9 (11.8)	77.5 (13.7)
ALT (u/L)^a^	Mean (SD)	30.6 (13.8)	27.1 (10.4)	65.7 (19.0)	26.9 (9.8)	34.4 (19.4)

^a^ Ns for ALT: 63 (MOD), 160 (MARD), 82 (SIRD), and 152 (SIDD). *N*: number of patients, *SD* Standard deviation, *MOD* Mild obesity-related diabetes, *MARD* Mild age-related diabetes, *SIRD* Severe insulin-resistant diabetes, *SIDD* Severe insulin-deficient diabetes, *HbA1c* hemoglobin A1c, *HOMA2-B and HOMA2-IR* Homoeostatic model assessment 2 estimates of β-cell function and insulin resistance, *BMI* Body mass index, *ALT* Alanine aminotransferase

### Stages of diabetes reversal

**Fig. 2:**
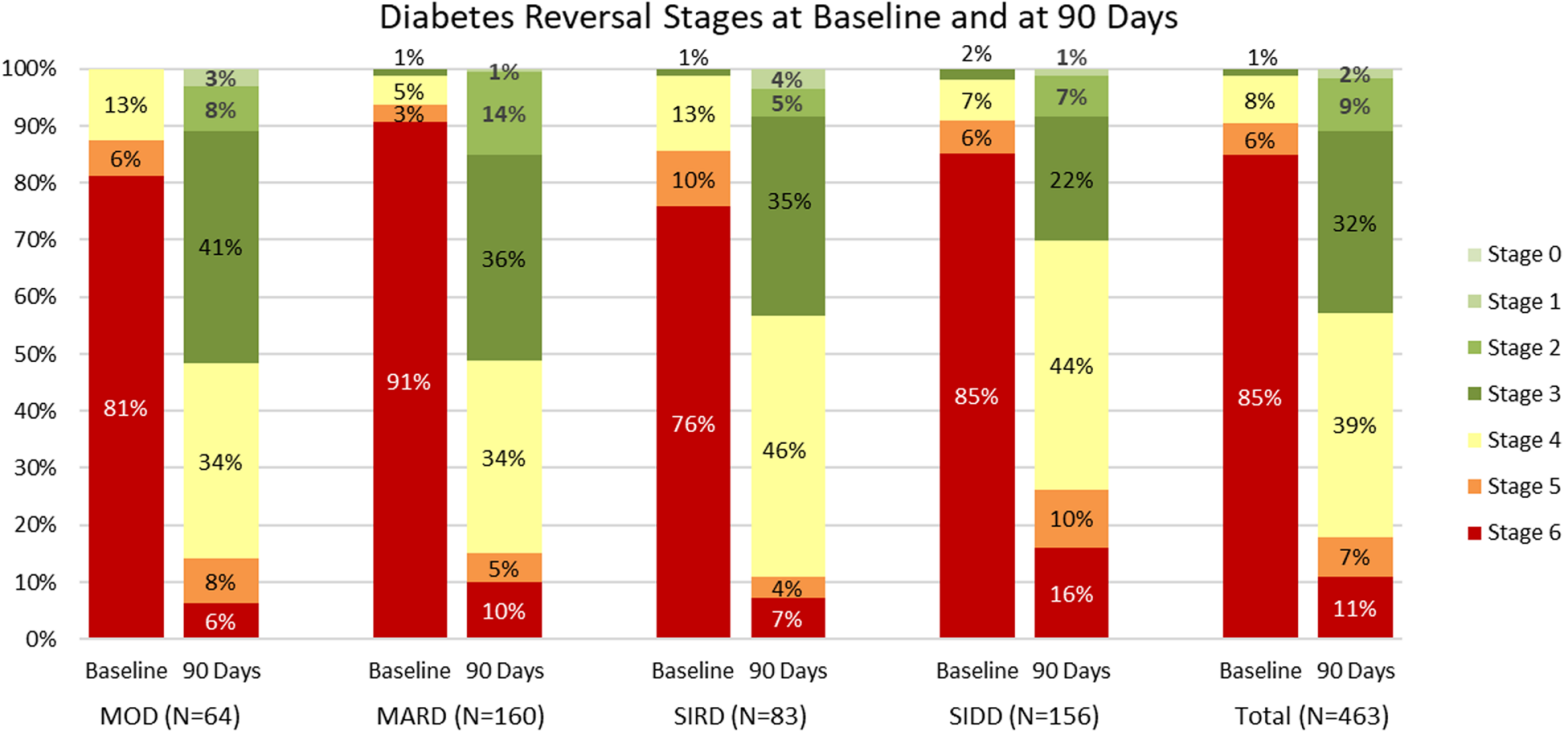
Stages of Diabetes Reversal for 463 Patients at Baseline and at 90 Days by Type 2 Diabetes Cluster. Diabetes reversal stages: Stage 6: > = 3 antidiabetic medications; Stage 5: dual oral therapy; Stage 4: metformin monotherapy (< 2000 mg) with eA1c between 5.7 and 6.4%; Stage 3: no antidiabetic medication with eA1c between 5.7 and 6.4%; Stage 2: HbA1c < 6.5% without antidiabetic medication; Stage 1: HbA1c < 5.7% without antidiabetic medication for less than 1 year; Stage 0: HbA1c < 5.7% without antidiabetic medication for more than 1 year. MOD: mild obesity-related diabetes; MARD: mild age-related diabetes; SIRD: severe insulin-resistant diabetes; SIDD: severe insulin-dependent diabetes

The change from baseline to 90 days in distribution across reversal stages is shown in Fig. 1 for this population and in Fig. 2 for each of the clusters. In total, 84.9% of these patients began in Stage 6 (at least three antidiabetic medications). After 90 days, only 11.0% remained at Stage 6, and 82.1% were at Stage 4 or better (metformin monotherapy [< 2000 mg] with eA1c between 5.7 and 6.4%). A Wilcoxon signed rank test showed that the change in distribution of reversal stages from baseline to 90 days for all patients was statistically significant (*p* < 0.0001). Additional tests showed that the change in distribution of reversal stages was also statistically significant in each of the clusters (all *p* < 0.0001).

### Survival analysis of time to reversal stage 3

**Fig. 3:**
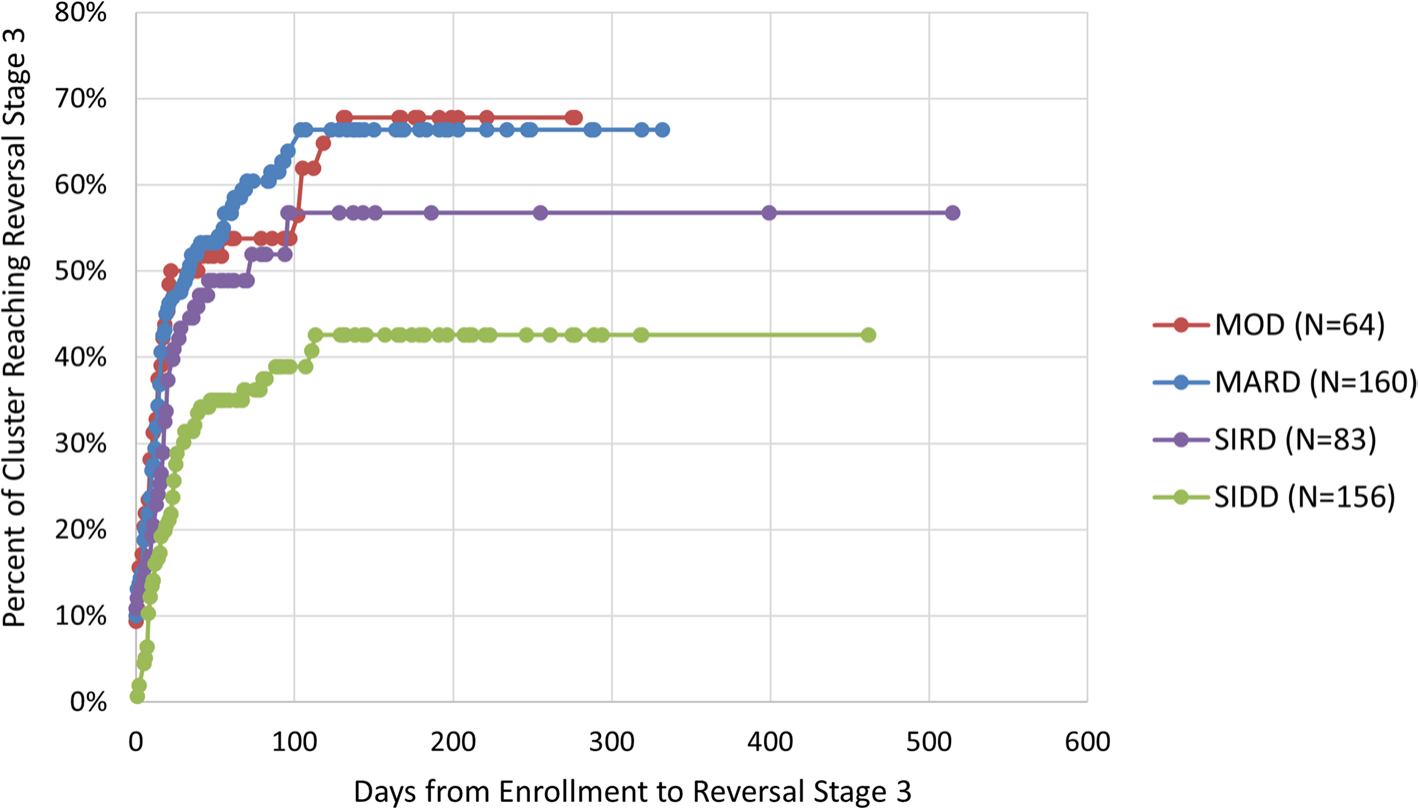
Days to Reversal Stage 3 for 463 Patients. Diabetes reversal Stage 3: no antidiabetic medication with eA1c between 5.7 and 6.4%. MOD: mild obesity-related diabetes; MARD: mild age-related diabetes; SIRD: severe insulin-resistant diabetes; SIDD: severe insulin-dependent diabetes

Taking censored values into account, the median number of days from a patient’s enrollment to reaching diabetes reversal Stage 3 (no antidiabetic medication with eA1c between 5.7 and 6.4%) was 80 days across all patients. The median time was shorter in MOD (41 days) and MARD (31 days) than in SIRD (73 days) and SIDD (value censored). Figure 3 shows the results of the Kaplan-Meier survival analysis of the number of days to reaching diabetes reversal Stage 3. By 90 days after enrollment, 59% of MARD, 53% of MOD, and 49% of SIRD had reached Stage 3, but only 37% of SIDD had reached that milestone. These four percentages were significantly different overall (*p* = 0.0010). In pairwise comparisons, the percentages of MARD and SIDD reaching Stage 3 by 90 days were significantly different from each other (*p* = 0.0005), and the percentages for MOD vs SIDD (*p* = 0.0698) and SIDD vs SIRD (*p* = 0.1086) were close to significantly different (*p*-values are corrected for multiple comparisons using the Benjamini-Hochberg correction). A log-rank test to test whether there were any pair-wise differences in overall progression between clusters (as shown in Fig. 3) found that MARD (*p* < 0.0001), MOD (*p* = 0.0023), and SIRD (*p* = 0.0425) were each significantly different from SIDD (p-values are corrected for multiple comparisons using the Benjamini-Hochberg correction).

### Baseline vs 90 days comparisons

Table 2 provides a comparison of mean (SD) values of a variety of clinical characteristics from patients with baseline and 90-day measurements available. Overall, there was a 21.2% decrease in average HbA1c over 90 days (*p* < 0.0001). HbA1c decreased significantly in all four clusters, with larger decreases in SIRD and SIDD. Across the whole population, the change in HOMA2-B was not significant, but HOMA2-B increased significantly in SIRD and SIDD and decreased significantly in MOD (yet after this decrease, HOMA2-B remained in the normal range in MOD patients). Reversal in MOD patients seems to be more dependent on reduction of HOMA2-IR than on increasing HOMA2-B. The overall 35.3% decrease in HOMA2-IR was significant (*p* < 0.001), and HOMA2-IR decreased significantly in all clusters except MARD. While the overall change in ALT was not significant, ALT decreased significantly in SIRD. BMI decreased 6.3% overall (*p* < 0.001) and decreased significantly (5.5 to 7.7%) in each cluster.

**Table 2:** Change in Clinical Metrics: Baseline vs. 90 Days

	N	Baseline Mean (SD)	90 Days Mean (SD)	% Change	***P***-value
**HbA1c (%)**	204	8.9 (1.8)	7.0 (0.9)	−21.2%	< 0.001
**HOMA2-B**	237	55.1 (38.6)	57.6 (33.3)	4.5%	0.37
**HOMA2-IR**	237	2.3 (1.7)	1.5 (0.8)	−35.3%	< 0.001
**ALT (u/L)**	239	32.5 (17.7)	30.7 (32.4)	−5.8%	0.39
**BMI (kg/m**^**2**^**)**	250	28.3 (4.9)	26.5 (4.6)	−6.3%	< 0.001
**Weight (kg)**	247	78.0 (14.5)	72.8 (13.3)	−6.7%	< 0.001
**10-Year ASCVD Risk**	179	12.2% (12.2%)	10.6% (11.0%)	−12.7%	< 0.001
**eGFR (mL/min/1.73m**^**2**^**)**	239	102.2 (18.3)	104.0 (15.3)	1.7%	0.03
**Total WBC Count (cells/mm**^**3**^**)**	242	7764.9 (1746.7)	7405.7 (1719.9)	−4.6%	< 0.001
**Testosterone (Male) (ng/dL)**	109	396.1 (183.1)	453.4 (165.4)	14.5%	< 0.001

*SD* Standard deviation, *HbA1c* Hemoglobin A1c, *ALT* Alanine aminotransferase, *BMI* body mass index, *HOMA2-B and HOMA2-IR* Homoeostatic model assessment 2 estimates of β-cell function and insulin resistance, *ASCVD* Atherosclerotic cardiovascular disease, *eGFR* estimated glomerular filtration rate, *WBC* White blood cell

Table 2 also provides baseline vs 90-day comparisons for additional metrics. Weight decreased 6.7% on average (*p* < 0.001). Ten-year ASCVD risk dropped nearly 13% (*p* < 0.001). The population’s eGFR values increased 1.7% (*p* = 0.03). WBC decreased significantly overall (4.6%, *p* < 0.001). Male patients had a 14.5% increase in testosterone (*p* < 0.001). Testosterone did not change significantly for females. Patients had a significant average decrease in GGT (30.3%, *p* < 0.001, *N* = 109). Creatinine decreased 3.1% (*p* = 0.003, *N* = 294). Triglycerides decreased 18.8% (*p* < 0.001, *N* = 244), and HDL increased 6.8% (*p* < 0.001, *N* = 244). Changes in VLDL and ApoB were not significant overall. No significant changes in platelet count or magnesium levels were found. However, albumin levels increased significantly (3.3%, *p* < 0.001, *N* = 294) and globulin levels decreased significantly (6.1%, *p* < 0.001, *N* = 295). Sodium levels increased by 0.8% (*p* < 0.001, *N* = 249), and potassium levels decreased 2.1% (*p* = 0.03). Chloride levels increased by 1.4% (*p* < 0.001).

## Discussion

The current study is the first to examine stages of diabetes reversal before and after 90 days of precision nutrition therapy. The distribution of patients across reversal stages changed significantly for the better during the first 90 days of participation in the TPN program for all clinically-defined patient subgroups combined as well as within each patient subgroup. At baseline, few patients (9.5%) were in reversal Stage 4 or better, but over the first 90 days, most patients (82.1%) reached advanced stages of reversal (Stage 4 or better) with improved clinical outcomes and fewer antidiabetic medications. At baseline, 90.5% of patients were taking at least two oral antidiabetic medications (Stages 5 and 6). By 90 days, only 17.9% remained in Stages 5 and 6, and 42.8% had eliminated all diabetes medications and had eA1c < 6.5% (Stage 3 or better). Additionally, there were significant differences in progression rates between subgroups of patients. The rate at which patients reached Stage 3 varied by cluster, with MOD and MARD patients reaching Stage 3 quickly and SIDD patients reaching Stage 3 more slowly.

Prior diabetes reversal studies have primarily defined reversal as a binary event that is reached when patients eliminate antidiabetic medications and maintain HbA1c < 5.7% for a year [3, 6–9, 14, 15]. In the Diabetes Remission Clinical Trial (DiRECT), Lean et al. found that 46% of patients in a year-long weight management program achieved diabetes remission (HbA1c < 6.5% at least 2 months after discontinuing all antidiabetic medications) [6]. A study by McInnes et al. found 40.7% of patients reached partial or complete diabetes remission (defined by fasting and 2-h plasma glucose measurements and use of diabetes drugs) 12 weeks after a 16-week intensive metabolic intervention [3]. Only one study was found that divided diabetes reversal into more levels than “partial” and “complete.” Mottalib et al. found that 22% of obese patients with diabetes less than 3 years had either major glycemic improvement (HbA1c < 6.5% with or without antihyperglycemic medications), partial remission (HbA1c 5.7–6.5% without antihyperglycemic medications), or complete remission (HbA1c < 5.7% without antihyperglycemic medications) 1 year after a 12-week weight management program [7]. The current study defined seven stages of diabetes reversal progression and found significant progression from higher to lower stages over the first 90 days of the TPN program. Viewing diabetes reversal in multiple stages may be more beneficial for patients by giving them short-term goals and milestones that indicate progress toward complete reversal.

In his review of the evidence for reversibility of diabetes [15], Ang cited the need for additional research into the subgroups of diabetes patients for whom reversal would be feasible and specifically mentioned the need for research in non-obese patients as well as the SIDD, SIRD, MOD, and MARD subgroups. The current study provides evidence that reversal progression can occur, particularly in the MARD, MOD, and SIRD clusters. It is also of note that the baseline BMI of the MARD and SIRD clusters was only 27 kg/m^2^, indicating that reversal progression can occur in non-obese populations.

The generalizability of the current study is limited by its retrospective nature, lack of control arm, and missing data metrics for some patients. Additionally, the study was not long enough to measure the percent of patients reaching diabetes reversal Stage 0 (HbA1c < 5.7% without antidiabetic medications for more than 1 year). Examining progression through diabetes reversal stages over a longer period of time is a subject of research that is already underway.

## Conclusions

This study defines seven stages of diabetes reversal for type 2 diabetes patients. This study provides evidence that diabetes reversal progression occurs in both non-obese and obese patients and in patients with longer duration of diabetes. Additionally, the study identifies clinically-defined subgroups of patients where progression occurs more quickly, depending on baseline characteristics of the subgroups. Use of reversal stages may lead to future specialization of milestone-oriented therapy and improve patient motivation during therapy, particularly for patients in subgroups with higher potential for remission.

## Data Availability

Data are available from the authors upon reasonable request.

## Abbreviations

HbA1c: Hemoglobin A1c
BMI: Body mass index
eA1c: Estimated HbA1c
ADA: American Diabetes Association
TPN: Twin Precision Nutrition
CGM: Continuous glucose monitors
ALT: Alanine aminotransferase test values
HOMA2-IR: Insulin resistance
HOMA2-B: Homoeostatic model assessment 2 estimate of β-cell function
HDL: High density lipoprotein
VLDL: Very low density lipoprotein
ApoB: Apolipoprotein B
GGT: Gamma-glutamyltransferase
eGFR: Estimated glomerular filtration rate
WBC: White blood cell
ASCVD: Atherosclerotic cardiovascular disease
SD: Standard deviation
MOD: Mild Obesity-related Diabetes
MARD: Mild Age-Related Diabetes
SIRD: Severe Insulin-Resistant Diabetes
SIDD: Severe Insulin-Dependent Diabetes
